# Radiotherapy-Associated Pain in Head and Neck Cancer: From Clinical Burden to Neuroimmune Modulator

**DOI:** 10.3390/jcm15135040

**Published:** 2026-06-28

**Authors:** Wenjun Meng, Ruiyue Li, Manting Wang, Zilin Yue, Haoran Zhang, Xueliang Sun, Qing Li

**Affiliations:** 1 Department of Pain Management, West China Hospital, Sichuan University, Chengdu 610041, China; mwj1995@scu.edu.cn; 2 Outpatient Department, West China Hospital, Sichuan University, Chengdu 610041, China; 3 West China School of Medicine, Sichuan University, Chengdu 610041, China; 4 Department of Biotherapy, Cancer Center, West China Hospital, Sichuan University, Chengdu 610041, China; 5 Department of Biotherapy, Cancer Center, National Clinical Research Center for Geriatrics, West China Hospital, Sichuan University, Chengdu 610041, China

**Keywords:** radiotherapy, radiotherapy-associated pain, neuroimmune crosstalk, head and neck cancer, immunotherapy, tumor immune microenvironment

## Abstract

Radiotherapy-associated pain is among the most common and debilitating complications in head and neck cancer. Although historically viewed primarily as a treatment-related adverse effect, growing evidence suggests that pain is deeply intertwined with tumor biology, immune remodeling, and therapeutic outcomes. At the same time, recent advances in cancer neuroscience have identified sensory nerves as active components of the tumor microenvironment (TME), capable of influencing antitumor immunity through complex neuroimmune crosstalk. These observations raise the possibility that radiotherapy-associated pain is not merely a clinical symptom but also a biological indicator of dynamic changes within the tumor immune microenvironment (TIME). In this review, we outline the major clinical manifestations of radiotherapy-associated pain in head and neck cancer, including inflammatory or mucositis-related pain, neuropathic pain, and long-term chronic pain, with emphasis on their underlying biological features and potential therapeutic relevance. Given that oral mucositis is the dominant source of acute radiotherapy-associated pain in head and neck cancer, we further summarize evidence-based preventive and supportive strategies, including photobiomodulation, mucosal barrier-forming agents, anti-inflammatory mouthwashes, nutritional interventions, pain control, and multidisciplinary oral care. We further discuss how radiotherapy reshapes the TIME through mechanisms such as immunogenic cell death, activation of the cGAS-STING pathway, vascular and stromal remodeling, and regulation of lymphoid compartments, while also triggering compensatory immunosuppressive responses. Preclinical and translational studies suggest that nociceptive signaling pathways may modulate T-cell function, myeloid-cell activity, and immune-evasive programs. Through these neuroimmune interactions, radiotherapy-induced neural injury and persistent pain may contribute to the establishment of an immunosuppressive, wound-like microenvironment that ultimately affects treatment response and tumor progression. Finally, we discuss the translational significance of incorporating pain phenotyping into combined radiotherapy and immunotherapy strategies for head and neck cancer. Opioid-sparing multimodal analgesia, neuromodulation, and neuroimmune-targeted interventions may represent promising approaches to simultaneously improve symptom control and antitumor immunity. We propose that radiotherapy-associated pain may be considered a candidate neuroimmune phenotype rather than a passive adverse event, providing a new conceptual framework for precision management and translational research in head and neck cancer.

## 1. Introduction

Pain is one of the most clinically visible and biologically complex features of head and neck cancer [1]. Because the head and neck region is densely innervated, patients often experience severe pain from the tumor itself as well as from treatment-related injury, such as oral mucositis (OM) and neuropathic pain post-radiotherapy [2,3,4]. In recurrent and metastatic head and neck squamous cell carcinoma (HNSCC), pain is sufficiently prevalent that opioid therapy remains a mainstay of symptom control. However, opioid exposure plus long-term radiation exposure may influence immune status and clinical outcomes [5,6]. This makes pain management more than a supportive-care issue: it may be part of the broader therapeutic context in which radiotherapy and immunotherapy operate [7,8].

At the same time, cancer neuroscience has fundamentally changed how we understand radiotherapy-related pain [9]. Traditional views regarded pain primarily as a consequence of tissue injury induced by tumor invasion or radiotherapy. However, accumulating evidence indicates that pain is itself an active biological process involving dynamic bidirectional communication between sensory neurons, immune cells, and tumor cells within the tumor microenvironment (TME) [10]. Peripheral nerves are now recognized as active components of the TME rather than passive conduits for nociception, capable of regulating immune surveillance, tumor progression, and therapeutic responses [11,12]. Through the release of neuropeptides, chemokines, cytokines, and other mediators, nociceptive signaling has been implicated in the modulation of the tumor immune microenvironment (TIME) in preclinical models and may be associated with reduced treatment response [13,14,15,16]. For example, radiation has been shown to induce HNSCC cells to secrete CCL20, which recruits regulatory T cells (Tregs) and suppresses antitumor immune responses, ultimately impairing radiotherapeutic efficacy [17]. Recent preclinical studies suggest that nociceptive neuroimmune circuits can contribute to immune-suppressive states; however, direct evidence in radiotherapy-treated HNSCC patients remains limited [18]. Collectively, radiotherapy-associated pain should not be viewed merely as a treatment-related toxicity but rather as a “neuroimmune phenotype” with emerging biological and therapeutic implications.

In this review, we propose that this conceptual framework may help explain interpatient heterogeneity in treatment response and reveal new translational opportunities for combining radiotherapy, immunomodulation, and pain-targeted interventions in head and neck cancer.

## 2. Clinical Phenotypes: Pain and Subtyping During/After Radiotherapy

Radiation-related pain is not a single symptom but a heterogeneous phenotype across time windows. Large longitudinal cohort studies have shown that pain peaked at 4 months post-diagnosis and remained significant at 12 months in head and neck cancer survivors, suggesting that pain is a persistent clinical problem rather than a transient side effect of treatment [19]. Therefore, radiotherapy-associated pain is not a static concept but a continuous process that evolves from tumor-related pain and treatment-related acute pain to chronic survivorship pain [20]. Mechanistically, radiation-related pain can be divided into at least three categories: (1) acute inflammatory pain, mainly related to OM and regional tissue damage; (2) neuropathic pain, mainly related to sensory nerve damage caused by radiation or tumor infiltration; and (3) persistent/chronic pain, which usually occurs long after radiotherapy and is often associated with fibrosis, local structural changes, nerve sensitization, and survivorship dysfunction [20,21]. This subtyping perspective is important because different phenotypes correspond to different molecular pathways, different risk factors, and different intervention strategies.

Classifying radiation-related pain is not merely for conceptual categorization but rather to guide clinical decision-making. Acute mucositis pain is better suited to local barrier protection, anti-inflammation, photobiological modulation, and nutritional support; neuropathic pain is better suited to pregabalin, neuromodulation, and multimodal analgesia; and persistent/chronic pain requires long-term follow-up, functional rehabilitation, and multidisciplinary pain management during the survivorship phase [2,22,23,24].

Furthermore, classifying radiation-related pain has significance for research. OM often co-occurs with treatment interruption, nutritional decline, weight loss, and infection risk, all of which further affect patients’ ability to receive subsequent treatment [22,25,26]. This means that pain is not only a symptom but also a mediating variable that may affect treatment sustainability and overall disease management. Future prospective studies incorporating pain subtypes, opioid exposure, nutritional status, and local inflammatory markers will be more likely to explain why different patients exhibit completely different clinical trajectories under similar radiotherapy regimens.

### 2.1. Oral Mucositis-Related Pain: The Main Phenotype of Pain During Radiotherapy

In radiotherapy for HNSCC, OM-related pain is the most prevalent pain phenotype and the leading cause of treatment interruption. Its clinical manifestations are characterized by mucosal erythema, erosion, ulceration and severe pain, which seriously interfere with the patient’s eating, drinking and speech functions, thereby significantly reducing quality of life (QoL) [27,28]. Nearly all HNSCC patients receiving radiotherapy develop OM of varying severity, which worsens alongside the cumulative radiation dose [22,29]. Low body mass index (BMI), low hemoglobin levels, poor oral hygiene, tumor site and concurrent chemoradiotherapy are all risk factors linked to severe OM [25,26]. This is why radiation-related pain often occurs concurrently with these manifestations rather than in isolation. Emerging evidence suggests oral protection, regional anti-inflammatory interventions, and photobiomodulation strategies can reduce pain and the need for analgesics [2,23,30].

OM is not only a local mucosal toxicity but also a central driver of radiotherapy-associated pain, nutritional compromise, opioid exposure, infection risk, and unplanned treatment interruption in patients with head and neck cancer. The updated MASCC/ISOO guidelines emphasize that OM is associated with pain, difficulty eating and swallowing, the need for enteral or parenteral nutrition, increased opioid consumption, and interruptions to cancer therapy [31]. Recent evidence summaries further report that radiotherapy-induced OM occurs in approximately 80–100% of patients with head and neck cancer receiving radiotherapy, with severe OM reported in 28–80%, depending on treatment intensity and patient risk factors [32]. Therefore, OM-related pain should be regarded as the dominant acute pain phenotype during radiotherapy rather than as a secondary supportive-care issue.

Mechanistically, radiation-induced OM evolves through epithelial injury, oxidative stress, inflammatory amplification, ulceration, and healing. Reactive oxygen species and pro-inflammatory pathways, including NF-κB, TNF-α, IL-1β, and IL-6, contribute to mucosal barrier breakdown and nociceptor sensitization, while ulceration increases the risk of secondary infection and further inflammatory pain [33]. These processes explain why mucositis pain frequently co-occurs with dysphagia, weight loss, malnutrition, dehydration, and increased analgesic requirements.

Current evidence-based management, therefore, requires a preventive and multidisciplinary approach. Core strategies include baseline dental and oral assessment before radiotherapy, standardized oral-care protocols, patient education, regular mucositis grading during treatment, nutritional risk screening, early dietitian involvement, topical/local pain control, and escalation to systemic analgesia when severe pain compromises oral intake. A recent evidence summary of OM management in head and neck cancer synthesized 22 best-evidence items across six domains: multidisciplinary management, oral assessment, basic oral care, pain management, nutritional support, and the application of honey or propolis [32].

### 2.2. Neuropathic Pain: Another Key Phenotype Emerging in Late or Post-Radiotherapy

Not all radiation-related pain stems from mucositis, but a significant proportion of patients experience radiotherapy-related neuropathic pain, whose clinical features often include burning, stabbing, electric shock-like, touch-evoked pain, or persistent facial and maxillofacial discomfort, suggesting sensory nerve damage or sensory pathway remodeling [20,21]. This type of pain differs from simple mucosal damage; it is typically more persistent and more likely to continue even after the local tissue surface has healed. A recent study showed a significant reduction in pain when pregabalin was added to treatment involving transcutaneous auricular vagus nerve stimulation (taVNS), suggesting that post-radiotherapy pain is not merely a matter of persistent inflammation but may be an independent subtype of pain that can be improved through neuromodulation [24].

### 2.3. Persistent/Chronic Pain: A Major Concern in Cancer Survivors

The end of radiotherapy does not mean the end of pain. Studies show that 35.5% of patients still experience significant pain 12 months after radiotherapy, and a considerable proportion of patients with head and neck-specific pain have not recovered below baseline [19]. For example, common post-radiotherapy symptoms such as trismus and dysphagia are mainly attributed to muscular fibrosis and nerve injury, which serve as major sources of chronic pain [34,35]. Such persistent pain substantially impairs patients’ QoL, including oral health-related QoL and general functional status [36,37]. Moreover, pain risk is closely related to oral cancer, advanced stage, smoking, depression, and comorbidities, indicating that persistent pain has shifted from acute toxicity to survivorship burden [19].

Chronic pain is likely the result of multiple factors, including local tissue fibrosis, tissue remodeling, persistent pain sensitization caused by nerve damage, temporomandibular joint dysfunction, alveolar bone and salivary gland damage, and the long-term use of analgesics. These pathological mechanisms are difficult to distinguish individually in a single clinical study, but existing systematic reviews have confirmed the prevalence of persistent pain after radiotherapy, necessitating targeted intervention studies [21].

Radiotherapy-associated pain in head and neck cancer should not be interpreted merely as a passive consequence of tissue injury. On the contrary, it may reflect, or coexist with, an active neuroimmune process in which nociceptive signaling, sensory nerve remodeling, and immune regulation are tightly interconnected within the TIME [38,39]. Persistent/neuropathic pain after radiotherapy may indicate sustained nociceptor activation, altered neuropeptide signaling, and ongoing remodeling of local immune function [40]. This perspective provides a mechanistic bridge between clinical pain phenotyping and downstream TIME reprogramming, and it supports the rationale for integrating analgesic strategies, neuromodulation, and immune-oriented treatment approaches in head and neck cancer [41].

Pain subtyping corresponds directly to distinct therapeutic strategies. Given the close correlation between pain and nutritional status, infection risk, and treatment adherence, this lays a solid clinical foundation for further exploring the mechanisms underlying pain-mediated regulation of tumor immunity.

## 3. How Radiotherapy Reshapes the TIME: From Cytotoxicity to Immune Reprogramming

**Figure 1:**
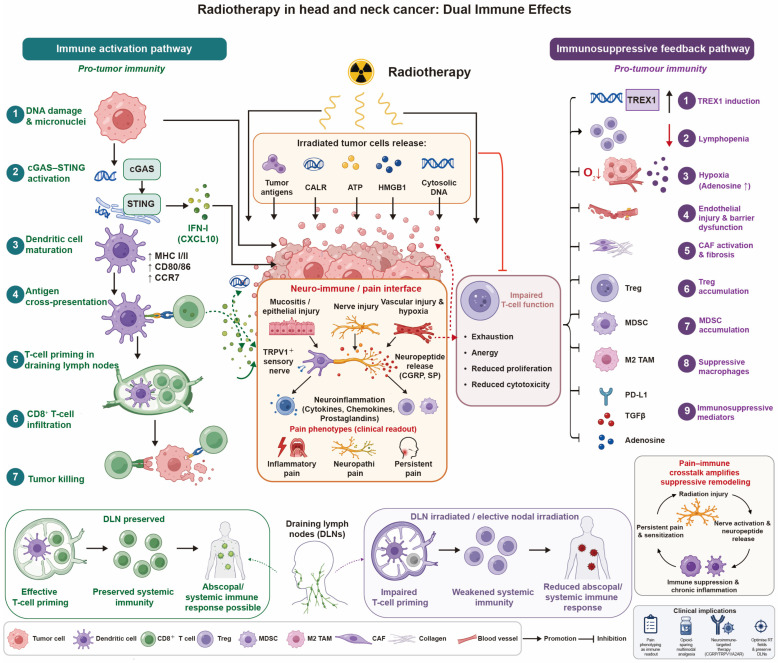
**Proposed model of radiotherapy-induced neuroimmune remodeling in head and neck cancer.** Radiotherapy can induce both immune activation and immunosuppressive remodeling within the TIME. DNA damage, cytosolic DNA accumulation, and immunogenic cell death may activate the cGAS-STING pathway, promote IFN-I signaling, DC maturation, antigen presentation, and CD8^+^ T-cell priming. Conversely, radiotherapy may also induce suppressive mechanisms, including TREX1 upregulation, lymphopenia, hypoxia, stromal remodeling, and accumulation of immunosuppressive cell populations and mediators. The central panel illustrates a proposed neuroimmune/pain interface whereby radiotherapy-induced mucosal injury, nerve damage, vascular dysfunction, and hypoxia may activate sensory nerves and neuropeptide signaling (e.g., CGRP and SP), potentially influencing immune cell behavior and contributing to neuroinflammation. These interactions are hypothesized to link radiotherapy-associated pain with dynamic changes in the TIME. At the systemic level, preservation of DLNs may support T-cell priming and systemic antitumor immunity, whereas elective nodal irradiation or DLN damage may impair immune initiation. Importantly, the neuroimmune pathways shown here represent a conceptual and hypothesis-generating framework derived from preclinical, translational, and indirect clinical evidence. Many of the proposed links have not yet been directly validated in patients with irradiated head and neck squamous cell carcinoma.

In head and neck cancer, radiotherapy-induced mucositis, local hypoxia, vascular changes, and inflammatory cell infiltration often occur concurrently with pain, dysphagia, and nutritional decline. In other words, radiotherapy-related pain is not a “side symptom” outside of immune remodeling but is very likely one of the clinical manifestations of the TIME being reprogrammed (Figure 1) [42].

**Figure 2:**
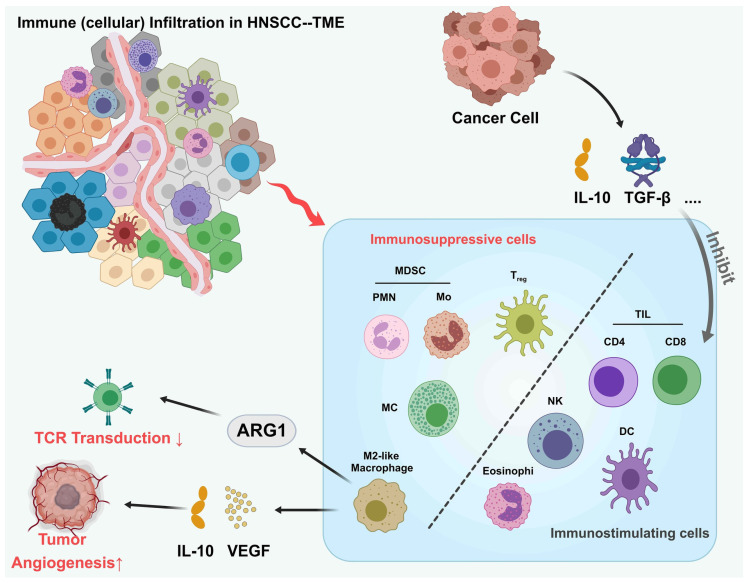
**Tumor immune infiltrating microenvironment of HNSCC.** The TME in HNSCC, highlighting interactions between immune cells and cancer cells. Tumor cells secrete immunosuppressive cytokines (IL-10 and TGF-β) that recruit myeloid-derived suppressor cells (MDSCs; PMN and Mo subsets), regulatory Tregs, M2-like macrophages, and eosinophils. These cells inhibit TILs, including CD4^+^, CD8^+^ T cells, NK cells, and DCs, suppressing antitumor immunity. ARG1 expression in MDSCs and M2-like macrophages further dampens TCR signaling. Additionally, VEGF secretion by tumor cells promotes angiogenesis. This balance between immunosuppressive and immunostimulatory cells shapes the TME, influencing tumor progression. (Reproduced with permission from Ref. [46]. 2025 Frontiers Media SA).

The TIME of head and neck cancer should be considered as part of a broader and dynamically evolving TME, rather than as an isolated immune compartment. Recent evidence emphasizes that the HNSCC TME consists of immune cells, cancer-associated fibroblasts (CAFs), extracellular matrix (ECM), hypoxic niches, metabolic gradients, and cytokine networks that interact bidirectionally with malignant cells and collectively shape immune evasion and therapeutic resistance (Figure 2) [43,44,45,46]. Špiljak et al. recently summarized this concept and highlighted that TME plasticity, spatial heterogeneity, CAF-mediated stromal remodeling, immune checkpoint dysregulation, hypoxia, and metabolic dependencies represent major barriers to durable treatment responses in HNSCC [47]. Therefore, radiotherapy-induced TIME remodeling may include both immunogenic activation and compensatory immunosuppressive adaptation, depending on tumor context, timing, radiation dose/fractionation, human papillomavirus (HPV) status, stromal architecture, and metabolic state.

### 3.1. Radiotherapy Induces Antigen Exposure and Danger Signals via Local Tissue Damage

The effects of radiotherapy on tumors go beyond directly inducing DNA damage and cell death. More importantly, it continuously reshapes the TIME, transforming what were originally cytotoxic events into immune-recognizable hazard signals. Tumor cells undergo apoptosis, necrosis, senescence, and mitotic catastrophe after radiation, accompanied by the release or exposure of immunogenic cell death (ICD)-related molecules, such as calreticulin (CALR), ATP, HMGB1, and type I interferon (IFN-I) signaling [48,49,50,51]. These signals collectively promote dendritic cell (DC) recruitment, maturation, and antigen cross-presentation, thereby transforming the local radiotherapy response into adaptive immune activation.

The significance of this process is that radiotherapy does not merely reduce tumor burden but also alters how the tumor is seen by the immune system. In other words, under certain conditions, radiotherapy can act as an “in situ vaccine”, enabling tumor antigens to be presented to the host immune system in a more immunogenic form [52]. Accordingly, acute inflammatory pain during radiotherapy may temporally overlap with maximal ICD-associated signaling, suggesting that pain phenotypes could partially mirror the balance between immune activation and suppressive recovery within the TIME [23,26,53,54].

ICD represents another mechanism through which radiotherapy may enhance antitumor immunity [55]. Unlike tolerogenic cell death, ICD is characterized by the exposure or release of damage-associated molecular patterns, including calreticulin, ATP, HMGB1, and tumor-associated antigens [56,57]. These signals promote DC uptake of tumor material, DC maturation, migration to draining lymph nodes, antigen cross-presentation, and priming of tumor-specific CD8^+^ T cells [58,59]. Therefore, inflammatory responses after radiotherapy should not be interpreted only as tissue-damaging or immunosuppressive; under appropriate conditions, they can also support the cancer–immunity cycle.

Nevertheless, ICD does not automatically translate into durable antitumor immunity [60]. Its immunologic consequences depend on the integrity of DC function, tumor antigenicity, local cytokine context, myeloid-cell composition, immune checkpoint expression, and the degree of concurrent tissue injury [59,60]. Thus, in HNSCC, OM-related inflammation, radiotherapy-induced ICD, and pain-related inflammatory signaling may have overlapping but not identical implications for antitumor immunity [61,62].

### 3.2. The cGAS/STING/IFN-I Axis Linking Radiotherapy-Induced Immune Activation

Among the pathways that reshape the TIME through radiotherapy, the cGAS-STING axis holds a central position [63]. Radiation-induced double-strand breaks in nuclear DNA, micronucleus formation, and mitochondrial DNA leakage can all be recognized by cytoplasmic nucleic acid sensors, thereby activating STING and driving the expression of inflammatory factors such as IFN-I and CXCL10, promoting DC maturation, enhancing antigen presentation, and driving CD8^+^ T-cell recruitment to the tumor site [49,51,52,64].

However, this immune activation is not linearly enhanced. High single-dose radiotherapy can induce TREX1 upregulation, which degrades cytoplasmic DNA, thereby weakening cGAS-STING-mediated immune activation [49,52,65]; however, the role of dose per fraction is still debated, and TREX1 data are derived primarily from preclinical models [66,67]. In contrast, moderately fractionated or intermediate-dose fractionation regimens are more likely to preserve nucleic acid sensing and IFN-I responses, thus having an advantage in immune synergy. This also explains why different doses, fractionations, and irradiation sequences lead to completely different immune outcomes.

Importantly, the inflammatory milieu generated during cGAS-STING activation overlaps substantially with pathways involved in nociceptor sensitization, supporting a potential biological connection between radiation-induced pain and innate immune activation.

### 3.3. Radiotherapy Simultaneously Boosts Immune Activation and Suppression

The effect of radiotherapy on the TIME exhibits a significant biphasic nature. On the one hand, it can enhance the visibility of tumor antigens and T-cell-mediated clearance by upregulating MHC-I, inducing damage-associated molecular patterns (DAMPs), activating pattern recognition receptors (PRRs), and promoting DC migration. On the other hand, it can also cause lymphopenia, persistent local inflammation, upregulation of PD-L1, enrichment of Tregs and myeloid-derived suppressor cells (MDSCs), and the induction of inhibitory cytokines and metabolic restriction, thus creating a reverse immunosuppressive feedback loop [50,51,52].

This dual effect is especially relevant in HNSCC. The baseline TIME in HNSCC is frequently characterized by impaired DC activity, limited T-cell infiltration, profound T-cell exhaustion, and strong immune evasion. Under these conditions, radiotherapy may either stimulate immune activation and transform an immunologically “cold” tumor into a “hot” one or further reinforce local immunosuppression, depending on factors such as the pre-existing immune context, radiation field size, dose and fractionation schedule, and the use of concurrent immunotherapy [42,68,69]. Persistent pain and ongoing neuroimmune sensitization may further tilt this balance toward immune suppression, promoting the maintenance of inhibitory TIME states and shifting transient antitumor immune activation toward long-term immunosuppressive remodeling.

### 3.4. Vessels, Matrix, and Myeloid Cells in the Remodeling of the TIME During Radiotherapy

Beyond tumor cells and lymphocytes, radiotherapy can also significantly change the state of tumor vascular endothelium, CAFs, and myeloid cells [70]. Low to moderate doses of radiotherapy can locally induce changes in adhesion molecule expression and vascular permeability, which in the short term facilitates effector T-cell entry into the tumor; however, higher doses or excessive irradiation areas may lead to endothelial damage, tissue hypoxia, and fibrosis, thereby limiting immune cell infiltration and promoting barrier formation dominated by suppressor macrophages, CAFs, and MDSCs [50,51,52,71].

This suggests that post-radiotherapy immune remodeling does not occur at the single-cell level but rather within the entire network of tumor cells, endothelial cells, stromal cells, myeloid cells, and lymphocytes. This is why the same radiotherapy prescription can produce completely different immune consequences in different patients. These observations also provide a potential mechanistic bridge between neurogenic inflammation and TIME remodeling, which will be further explored through the roles of calcitonin gene-related peptide (CGRP) and other neuroimmune mediators.

### 3.5. Stromal, Hypoxic, and Metabolic Remodeling as Barriers to Antitumor Immunity After Radiotherapy

In addition to direct effects on tumor cells and lymphocytes, radiotherapy may reshape stromal and metabolic niches that influence immune accessibility and therapeutic resistance [72]. In HNSCC, CAFs produce dense collagen networks, fibronectin, tenascin-C, and other ECM components, generating a desmoplastic barrier that restricts immune cell infiltration and drug penetration [47,73]. CAF-derived cytokines and chemokines, including TGF-β, IL-6, and CXCL12, can further promote Treg and MDSC accumulation, exclude cytotoxic lymphocytes, and reinforce immune suppression [74,75]. Hypoxic niches represent another major layer of resistance: hypoxia-inducible factor (HIF)-dependent signaling promotes angiogenesis, glycolysis, lactate accumulation, immune escape, and radioresistance [76]. These stromal, hypoxic, and metabolic features provide a mechanistic rationale for combining radiotherapy with TME-modulating strategies, including TGF-β blockade, CXCR4 antagonism, IL-6 inhibition, hypoxia-targeted agents, metabolic inhibitors, and immune checkpoint combinations (Table 1) [77].

### 3.6. Draining Lymph Nodes Govern Radiotherapy’s Shift from Local Therapy to Systemic Immunity

Whether radiotherapy can induce systemic antitumor immunity largely depends on whether the tumor-draining lymph nodes (DLNs) are effectively preserved. DLNs are key sites for primate T-cell initiation, DC antigen presentation, and epitope spreading; therefore, once these areas are included in the irradiation field, systemic immunity is often weakened [52,65]. Existing studies have shown that while elective nodal irradiation (ENI) helps with regional control, it may impair T-cell initiation and distant immune responses [52,65]. Conversely, preserving some lymphoid structures, employing more precise field design, or delaying lymph node irradiation may better maintain the synergistic effect of radiotherapy and immunotherapy.

Therefore, the immunologic value of radiotherapy depends not only on the irradiation dose itself but also on whether the irradiation field preserves the anatomical structures required for systemic immune initiation.

The abscopal effect is a rare, explicit result of the above mechanism operating in its entirety [78,79]. Mechanistically, the abscopal effect is not a routine outcome of radiotherapy but rather a rare phenotype resulting from the convergence of the aforementioned steps [80,81]. Only when tumor antigen release, DC maturation, T-cell activation in DLNs, effector T-cell migration, and local immunosuppression are sufficiently overcome can local irradiation be transformed into suppression of distant, unirradiated lesions. For this reason, the abscopal effect suggests that radiotherapy is not only a local treatment tool but may also influence the trajectory of systemic disease through the immune system.

Therefore, radiation-associated pain is not simply a parallel toxicity but a clinically relevant factor that may influence systemic immune initiation through its effects on treatment continuity, nutritional decline, and inflammatory stress.

## 4. Core Mechanism: How the Pain–Neural–Immune Axis May Drive Changes in the TME

The mechanisms discussed in this section should be interpreted according to the strength and context of available evidence. Some mechanisms, such as radiotherapy-induced ICD, cGAS-STING activation, and compensatory immunosuppressive remodeling, are supported by broad radiobiological and tumor-immunology evidence. Other pathways, including CGRP-RAMP1 and adenosine A_2A_ receptor (A_2A_R) signaling, have stronger support in preclinical oral cancer models, HNSCC patient-derived immune cell assays, or non-radiotherapy settings. By contrast, the specific proposition that radiotherapy-associated pain actively promotes immune escape in HNSCC patients remains a hypothesis that requires prospective clinical validation.

**Figure 3:**
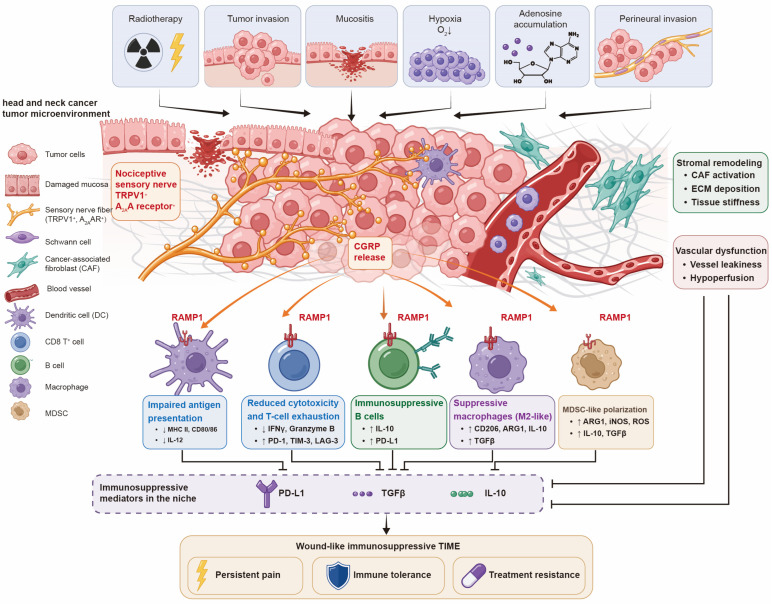
**Proposed CGRP-centered neuroimmune model linking nociceptive signaling and immunosuppressive remodeling in head and neck cancer.** Multiple stressors associated with head and neck cancer, including radiotherapy, tumor invasion, mucositis, hypoxia, adenosine accumulation, and perineural invasion, may activate TRPV1^+^ and A_2A_R^+^ sensory nerves and promote CGRP release within the TME. Through signaling pathways involving RAMP1, CGRP has been implicated in the regulation of both innate and adaptive immune responses. Experimental studies suggest that CGRP may impair dendritic-cell antigen presentation, promote T-cell dysfunction, and favor immunosuppressive phenotypes among B cells, macrophages, and myeloid populations. CGRP-associated neuroimmune signaling may also contribute to stromal remodeling and vascular dysfunction, potentially reinforcing immune exclusion and local immune suppression. Collectively, these interactions are hypothesized to support the development of a wound-like immunosuppressive TIME characterized by persistent pain, immune tolerance, and treatment resistance.

Pain is not a phenomenon independent of the TME but rather an outward phenotype resulting from the remodeling of sensory nerves, neuropeptides, immune cells, and stromal cells [82,83,84]. This axis is particularly active in high-density nerve-innervated regions like HNSCC; tumor invasion, radiation damage, mucositis, and neural reprogramming can amplify each other, forming a continuous neuroimmune feedback loop (Figure 3) [85,86].

The stromal compartment may provide an anatomical and molecular bridge between neural signaling and immune remodeling. CAFs and ECM remodeling can regulate tissue stiffness, cytokine gradients, immune cell trafficking, and therapeutic penetration, while inflammatory mediators produced in irradiated tissues may sensitize nociceptors and reshape local immune responses. Although direct evidence in irradiated HNSCC remains incomplete, integrating stromal remodeling into the neuroimmune framework helps explain how treatment-induced tissue injury, pain-related inflammatory signaling, and immune exclusion may coexist within the same evolving TME.

### 4.1. From Radiation Damage to Neural Reprogramming: Pain as a Signal of a Rewritten Microenvironment

The local microenvironment of HNSCC is naturally rich in sensory, sympathetic, and parasympathetic nerve fibers. These nerves do not passively traverse between tumors but participate in tumor growth, invasion, and immune evasion. Common neurological events in HNSCC include perineural invasion (PNI), axonogenesis, and nerve reprogramming [85]. Furthermore, HPV-negative HNSCCs typically have higher nerve density and more pronounced pain, suggesting a close coupling between nerve load and clinical symptoms [87].

Radiotherapy acts as an additional modifier of these neurobiological processes [88,89]. On the one hand, it can damage tumor cells and alter their metabolism and immune phenotype; on the other hand, it can also damage normal tissues, exacerbate mucositis, and induce persistent pain and neurosensitization, thus pushing local tissues into a state similar to “chronic wound repair”. Persistent pain after radiotherapy for HNSCC indicates that this type of pain is not uncommon, and the evidence level for truly effective treatments remains limited, suggesting that it is not a transient side effect but a continuous biological process [21].

### 4.2. Sensory Nerves Are Active Regulators of Tumor Immunity

Mounting evidence suggests that sensory nerves can directly regulate tumor-infiltrating lymphocytes (TILs) and adaptive immune responses [14]. In HNSCC, CGRP released by sensory nerves directly inhibits CD8^+^ T-cell activity, while blocking sensory nerve function increases CD8^+^ and CD4^+^ T-cell activity and inhibits tumor growth [14]. Therefore, pain-related neural activity is not merely a companion phenomenon but may itself constitute an immunosuppressive circuit.

Further study indicates that increased nerve density in HNSCC is associated with an immunosuppressive TME phenotype; the richer the CGRP^+^ sensory nerves, the more pronounced the tumor’s immune escape characteristics [14,90]. Mechanistically, neuropeptides released by sensory nerves not only affect blood vessels and the matrix but also directly alter the activation state of T cells, DCs, and myeloid cells, creating a local environment biased towards a low-reactivity, low-kill, and high-repair state [13,91].

### 4.3. The CGRP-RAMP1 Axis: Linking Pain Neuropeptides to Immune Escape

CGRP is both a classic pain-related neuropeptide and a powerful immunomodulatory signal. CGRP release from sensory nerves can promote tumor growth, while inhibiting CGRP or sensory nerve function can enhance CD8/CD4^+^ T-cell activity and reduce tumor progression [14]. The CGRP-RAMP1 axis can directly regulate adaptive immunity: in skin microbiome-associated immunity, CGRP restricts type 17 responses through RAMP1, while upregulation of RAMP1 in T cells makes them direct target cells for neural signals [13].

In models such as HNSCC and melanoma, single-cell data suggest that CD8^+^ T cells exhibit RAMP1 upregulation, indicating that adaptive immune cells in the TME may already possess the ability to read neural signals [13,40]. That is, pain-related neuropeptides do not bypass the immune system to act indirectly but can directly set functional thresholds, activation thresholds, and exhaustion tendencies at the T-cell level.

It is worth noting that the effects of CGRP are significantly context-dependent. In tissue repair scenarios, it can promote healing through neutrophils and macrophages, but in tumor scenarios, the same repair–inflammation process may be hijacked by the tumor, transforming into a mechanism that promotes angiogenesis, restricts TIL entry, and maintains immune tolerance [92]. That is, CGRP is not simply a pro-inflammatory or anti-inflammatory molecule but a neuroimmune switch that determines the direction of immunity based on the tissue environment [14,92].

### 4.4. TRPV1, A_2A_ Receptor and Hypoxic/Adenosine Microenvironment: Coupling of Pain Signals and Metabolic Stress

Head and neck cancers, especially oral squamous cell carcinoma (OSCC), are often characterized by a coexistence of hypoxia, necrosis, adenosine accumulation, and chronic inflammation. In the adenosine-rich microenvironment of OSCC, TRPV1-positive nociceptive nerves can be stimulated by A_2A_R and release α-CGRP, thereby promoting tumor progression; clinically available A_2A_R antagonists can block this neurotumor crosstalk [85,93,94,95]. This finding links metabolic stress, neural excitation, and immune escape into a continuous chain.

This mechanism is particularly noteworthy in the context of radiotherapy. Post-radiotherapy, local tissues often experience transient hypoxia, cellular debris accumulation, and adenosine metabolic reprogramming [85,96]. These changes may increase the intensity of A_2A_R-related signals, thereby enhancing the tendency of sensory nerves to release CGRP. Direct evidence linking this pathway to radiotherapy-induced immune remodeling in HNSCC remains limited. Although further evidence is needed to directly prove the radiotherapy-A_2A_R-CGRP-immune escape pathway, mechanistically, this pathway is consistent with the biological environment after head and neck cancer radiotherapy.

### 4.5. Perineural Invasion, Schwann Cells, and the Neural–Mesenchymal–Immune Ternary Remodeling

Nerves in head and neck cancers are not isolated structures but rather constitute a dynamic ecosystem together with Schwann cells, the stroma, cancer cells, and immune cells [85]. Tumor cells can drive nerve growth, neural reprogramming, and PNI through molecules such as NGF, BDNF, GDNF, and galanin. Simultaneously, Schwann cells can be educated by the tumor to exhibit a repair-like phenotype, promoting ECM degradation, tissue remodeling, and tumor cell migration. This means that pain and nerve invasion often do not reflect abnormalities in a single neuron but rather indicate a complete reconfiguration of the neuro-mesenchymal network.

At the immunity level, PNI and nerve damage further weaken antitumor immunity. On the one hand, nerve damage can trigger local inflammation and immune cell rearrangement; on the other hand, PNI-related tissue remodeling makes tumors more likely to form wound-like niches, thereby increasing immune tolerance and the risk of treatment resistance [85]. This can also explain why clinically PNI-positive patients often experience more severe pain and poorer local control and are more prone to aggressive behavior.

### 4.6. From Nerve Damage to Immunosuppression: Pain May Be a Clinical Indicator of TME Being Neurologized

Combining the above evidence yields a clearer chain of events: radiotherapy and the tumor itself jointly cause nerve damage and sensory nerve excitation; the excited sensory nerves release neuropeptides such as CGRP; neuropeptides alter the state of T cells, B cells, DCs, and myeloid cells through receptors such as RAMP1 and A_2A_R, ultimately leading to an immunosuppressive microenvironment characterized by reduced TILs, decreased effector function, and enhanced TGFβ/PD-L1-related inhibition [13,14,97]. This chain is particularly compelling in esophageal squamous cell carcinoma (ESCC): CGRP-stimulated RAMP1^+^ B cells exhibit an immunosuppressive phenotype and weaken CD8^+^ T-cell killing, while RAMP1 blockade combined with anti-PD-1 partially restores antitumor immunity [97]. Although these findings derive from ESCC models, they support the possibility that similar CGRP-RAMP1-mediated neuroimmune circuits may exist in HNSCC.

Based on the above mechanisms, we can understand radiation-related pain as a clinical indicator: it not only reflects mucositis and nerve damage but may also suggest that the local TME has entered a state of high inflammation, high repair, and low killing. Significant changes in metabolism, apoptosis, and immune infiltration in tumor tissue after radiotherapy suggest that radiotherapy is indeed reshaping the local niche [98]. Therefore, when patients experience intractable pain, persistent mucositis, or neuropathic pain during radiotherapy, it is not only a matter of pain management but may also indicate that their local neural–immune axis is being continuously activated. Radiation-related pain is not a byproduct of tumor immune changes but may be a visible sign that neuroimmune reprogramming is underway. Table 2 summarizes the current evidence hierarchy for links among radiotherapy-associated pain, nociceptive signaling, and TIME remodeling in head and neck cancer.

## 5. Clinical Translation: How Pain Phenotypes May Help Refine Patient Stratification Strategies in Future Radioimmunotherapy Trials

The translational implications should be interpreted according to their current level of clinical readiness (Table 3). Established clinical management of radiotherapy-associated pain in head and neck cancer remains centered on evidence-based supportive care, including OM prevention and treatment, nutritional support, multimodal analgesia, rehabilitation, and multidisciplinary symptom management. By contrast, neuromodulation, CGRP/RAMP1 pathway targeting, and pain-guided immunotherapy represent emerging translational concepts rather than established clinical practices. Although early clinical and preclinical studies support their biological plausibility, direct evidence demonstrating durable clinical benefit, optimal patient selection, safety in combination with radiotherapy or immunotherapy, and effects on antitumor immunity remains limited. Therefore, these strategies should be framed as investigational opportunities that require prospective validation.

Radiation-related pain may not be best understood solely as a side effect; it may represent a clinically accessible phenotype for future neuroimmune stratification. In head and neck cancer, different pain phenotypes correspond to different tissue damage patterns, immune consequences, and analgesic strategies [29,107]. Therefore, to truly achieve synergy between radiotherapy and immunotherapy, the first clinical step is not to blindly increase treatment intensity but to incorporate pain subtypes, opioid exposure, mucosal toxicity, and immune readout into a stratification and decision-making framework [108,109].

Different pain phenotypes correspond to different combined treatment strategies. Table 4 presents the typical clinical features of different pain phenotypes, the biological states they are more likely to reflect, and their implications for combined radiotherapy/immunotherapy in head and neck cancer.

### 5.1. Evidence-Based Prevention and Management of Oral Mucositis-Related Pain

Because OM represents the leading acute pain phenotype during head and neck radiotherapy, its prevention and management should be embedded into combined radiotherapy, immunotherapy, and analgesic strategies [115]. The MASCC/ISOO clinical practice guidelines provide the most widely cited evidence-based framework for mucositis management and recommend or suggest several interventions in specific clinical settings, including multiagent oral-care protocols, benzydamine mouthwash, intraoral photobiomodulation (PBM), topical morphine mouthwash for OM-associated pain, oral glutamine in patients receiving radiochemotherapy, and honey in patients receiving radiotherapy or radiochemotherapy for head and neck cancer [31].

PBM has accumulated particularly strong evidence in head and neck radiotherapy. The MASCC/ISOO guidelines recommend intraoral PBM using low-level laser therapy for the prevention of OM in adults receiving radiotherapy to the head and neck and also in adults receiving radiochemotherapy for head and neck cancer, provided that validated treatment parameters are followed [31]. PBM serves as a non-invasive supportive-care intervention that alleviates inflammation, relieves pain and mitigates tissue injury arising from oral complications induced by cancer therapies [116]. Thus, when equipment and trained personnel are available, PBM should be considered as part of a proactive OM prevention pathway rather than reserved only for severe established mucositis.

Barrier-forming and mucoadhesive agents provide another clinically relevant approach by protecting ulcerated mucosa, reducing mechanical irritation, and relieving pain during swallowing and speaking. Recent clinical evidence on bioadhesive barrier-forming gels in head and neck cancer patients undergoing radiotherapy suggests that these agents can delay progression to advanced OM, reduce oral pain, and improve nutritional status and quality of life [22]. However, the strength of evidence varies across products; therefore, these agents are best described as supportive adjuncts rather than disease-modifying treatments.

Anti-inflammatory strategies are also relevant because mucositis is driven by inflammatory amplification. The MASCC/ISOO guidelines recommend benzydamine mouthwash for the prevention of OM in patients with head and neck cancer receiving moderate-dose radiotherapy and suggest its use in patients receiving radiochemotherapy [31]. Other anti-inflammatory agents, including corticosteroid-based mouthwashes, have emerging evidence but should be presented cautiously because their indications, dose, duration, and infection-related safety considerations require further validation in radiotherapy-treated HNSCCs.

Nutritional intervention should be integrated early, not delayed until severe mucositis develops. Severe OM-related pain impairs oral intake and can precipitate malnutrition, dehydration, treatment interruption, and hospitalization. Retrospective studies of early nutritional intervention in head and neck cancer radiotherapy indicate that nutritional risk screening, oral nutritional supplements, enteral feeding when swallowing is inadequate, and protein/calorie target achievement may ameliorate mucositis severity and maintain treatment tolerance [33]. A recent evidence summary likewise recommends dynamic nutritional risk assessment and individualized nutritional intervention, including oral supplements for patients who can eat and enteral or parenteral support when oral intake is insufficient [32].

Overall, OM-related pain management should be multidisciplinary [117]. Radiation oncologists, dentists or oral medicine specialists, nurses, dietitians, pharmacists, speech/swallowing therapists, and pain specialists should jointly monitor mucosal injury, pain intensity, oral intake, infection, weight loss, and treatment adherence. This multidisciplinary model is particularly important in HNSCC because uncontrolled mucositis pain may increase opioid exposure, impair nutritional status, and compromise the continuity of radiotherapy.

### 5.2. The Valuable Clinical Translational Direction: Opioid-Saving Multimodal Analgesia

For radiotherapy-induced mucositis pain, multimodal analgesia is a better priority than opioid monotherapy. The OPTIMAL-HN randomized clinical trial showed that in head and neck cancer patients receiving radical radiotherapy/chemoradiotherapy who developed moderate to severe mucositis pain, multimodal analgesia (pregabalin, acetaminophen, and naproxen, with opioids added if necessary) was non-inferior to opioid monotherapy in pain control during the last week of radiotherapy; and when analyzed over the full time window from the end of radiotherapy to 6 weeks, multimodal analgesia was even superior to opioid monotherapy [112]. The translational significance of this study is very clear: if we want to minimize the potential interference of opioids with immunotherapy, we should prioritize opioid-saving strategies rather than passively increasing the dosage after pain becomes uncontrollable.

When opioids are unavoidable to use, the problem becomes how to minimize immunosuppression while providing analgesia. McIlvried et al. suggest that morphine mediates immunosuppression through the μ-opioid receptor on CD8^+^ T cells and weakens anti-PD-1 efficacy, while peripherally acting μ-opioid receptor antagonists (PAMORAs) can reverse this suppression, theoretically preserving central analgesia [114]. This implies that opioid-sparing or peripheral-restriction analgesia is a clinical pathway worthy of further investigation in patients requiring long-term analgesia and preparing for immune checkpoint inhibitors (ICIs) [111].

### 5.3. Neuropathic Pain: The Approach Should Be Upgraded from Analgesia to Neuromodulation

The successful taVNS combined with pregabalin in patients with moderate to severe radiotherapy-related neuropathic pain suggests that neuropathic pain should be separated from mucositis pain in clinical practice and managed using different approaches [24,110]. From a mechanistic perspective, these patients warrant further investigation in trials including neuroimmunological markers, such as pain scores, along with simultaneous monitoring of neuropeptide pathways, T-cell function, and local inflammatory status. This is because if pain is indeed an overt manifestation of neuroimmunosuppression, then neuromodulation may not only relieve pain but also influence the TIME [10,108].

### 5.4. Pain Phenotypes Can Also Be Used in Trial Design of Radiotherapy/Immunotherapy and Patient Stratification

Valuable translational research will not involve lumping all patients together to determine combined benefit but rather pre-stratifying them by pain phenotype. For example: (1) Baseline stratification: Recording pain type, NRS/BPI, swallowing function, nutritional status, risk of oral mucositis, and prior opioid exposure before radiotherapy; (2) Dynamic stratification during treatment: recording mucositis grade, neuropathic symptoms, morphine milligram equivalent (MME), infection, and weight changes weekly; (3) Immune readout: simultaneous monitoring of CD8 TILs, DC function, PD-L1, inflammatory factors, and potential neural pathway markers in conjunction with pain management; and (4) Radiation parameters incorporated into the model, including irradiation volume, fractionation, and whether ENI is performed, as irradiation field design itself affects local and systemic immune responses [5,108,114,118,119]. ENI is not merely a technical parameter; it can influence systemic immune initiation and distant immune responses [118,120]. Therefore, in combined radiotherapy + immunotherapy studies, ENI should be treated as an important covariate, not background noise.

## 6. Limitations of Current Studies

This review proposes an integrative neuroimmune framework linking radiotherapy, nociceptive signaling, and tumor immune remodeling in head and neck cancer. However, several limitations should be noted. First, most mechanistic evidence is derived from preclinical models or other types of tumors, and direct clinical validation of radiotherapy-induced neuroimmune circuits, particularly CGRP-mediated pathways, remains limited. Second, the effects of radiotherapy on immune responses are highly context-dependent, influenced by dose, fractionation, irradiation volume, and baseline tumor immune status, introducing substantial heterogeneity across studies. Third, pain in HNSCC is multifactorial and reflects tissue injury, inflammation, infection, and psychological factors and should not be interpreted as a standalone marker of immune status.

In summary, the CGRP-centered neuroimmune model should be considered hypothesis-generating and requires prospective validation in clinical head and neck cancer cohorts integrating immune and neurobiological profiling.

## 7. Conclusions and Perspectives

In summary, radiotherapy-associated pain in head and neck cancer should no longer be viewed solely as a treatment-related symptom but rather as a biologically meaningful process linked to tumor–immune interactions. Increasing evidence supports an integrated model in which both radiotherapy and the tumor itself induce neural injury and sensory nerve activation, leading to local TIME remodeling through neuropeptide release and adenosine-mediated signaling. This process may ultimately establish a self-perpetuating cycle characterized by persistent pain, immune suppression, and heterogeneous therapeutic responses.

The key to future research is not simply pursuing a “reduction in pain scores” but rather answering the question: can we truly sever this circuit through more precise radiation therapy, more rational analgesia, and more targeted neuroimmune interventions, thereby simultaneously improving symptom control, treatment completion, and antitumor immune responses? This will be the core direction for radiation-related pain research to move from supportive treatment to mechanistic translation.

## Figures and Tables

**Table 1:** Dynamic remodeling and therapeutic vulnerabilities of the HNSCC TME after radiotherapy.

TME Component	Dynamic Remodeling/Radiotherapy-Relevant Change	Immune Consequence	Emerging Therapeutic Target or Strategy
**Tregs/MDSCs/TAMs**	Treatment-induced or tumor-driven enrichment of immunosuppressive populations	Suppression of CTLs, antigen presentation impairment, immune escape	Treg/MDSC/TAM targeting, checkpoint combinations
**Immune checkpoints**	Upregulation of PD-1/PD-L1, CTLA-4, LAG-3, TIM-3, TIGIT in exhausted immune cells	T-cell exhaustion and reduced antitumor immunity	PD-1/PD-L1 plus LAG-3/TIM-3/TIGIT combinations
**CAFs**	CAF activation, myCAF/iCAF heterogeneity, TGF-β/IL-6/CXCL12 signaling	Immune exclusion, Treg/MDSC recruitment, ICI resistance	TGF-β blockade, CXCR4 antagonists, IL-6 inhibition, CAF reprogramming
**ECM**	Collagen/fibronectin accumulation, matrix stiffening	Physical barrier to immune infiltration and drug delivery	ECM modulation, FAP targeting, improved drug delivery
**Hypoxia**	HIF-1α activation, VEGF induction, hypoxic niches	PD-L1 induction, impaired immune infiltration, radioresistance	Hypoxia-activated prodrugs, anti-angiogenic strategies, HIF targeting
**Metabolic remodeling**	Glycolysis, lactate accumulation, glutamine dependence	Acidic TME, suppressed CD8^+^ T/NK activity, Treg support	LDHA/glycolysis/glutaminase inhibitors, immunometabolic combinations
**Drug delivery barriers**	Dense ECM, abnormal vasculature, hypoxia, interstitial pressure	Poor penetration of chemo/immunotherapy	Nanoparticles, hydrogels, oncolytic virotherapy, local delivery

**Abbreviations:** TME, tumor microenvironment; Tregs, regulatory T cells; MDSCs, myeloid-derived suppressor cells; TAMs, tumor-associated macrophages; CTLs, cytotoxic T lymphocytes; PD-1, programmed cell death protein 1; PD-L1, programmed death-ligand 1; CTLA-4, cytotoxic T-lymphocyte-associated protein 4; LAG-3, lymphocyte activation gene 3; TIM-3, T-cell immunoglobulin and mucin domain-containing protein 3; TIGIT, T-cell immunoreceptor with Ig and ITIM domains; ICI, immune checkpoint inhibitor; CAFs, cancer-associated fibroblasts; myCAFs, myofibroblastic cancer-associated fibroblasts; iCAFs, inflammatory cancer-associated fibroblasts; TGF-β, transforming growth factor-β; IL-6, interleukin-6; CXCL12, C-X-C motif chemokine ligand 12; CXCR4, C-X-C chemokine receptor type 4; ECM, extracellular matrix; FAP, fibroblast activation protein; HIF-1α, hypoxia-inducible factor-1α; VEGF, vascular endothelial growth factor; LDHA, lactate dehydrogenase A; NK cells, natural killer cells.

**Table 2:** Evidence hierarchy for links among radiotherapy-associated pain, nociceptive signaling, and TIME remodeling in head and neck cancer.

Evidence Levels	Existing Evidence	References
**Established clinical evidence**	Radiotherapy-related pain, oral mucositis pain, and chronic pain in HNSCC are common and affect treatment adherence and quality of life; HNSCC also presents with immune escape and immunosuppressive TME.	[42]
**Radiobiology evidence**	Radiotherapy can enhance immune activation through ICD, cGAS-STING, IFN-I, and antigen presentation, and can also induce immunosuppressive feedback such as Treg, MDSC, PD-L1, TGF-β, hypoxia, and fibrosis.	[99,100]
**HNSCC-specific preclinical evidence**	Radiotherapy induces HNSCC tumor cells to secrete CCL20, recruits CCR6^+^ Tregs, suppresses antitumor immunity, and reduces the efficacy of radiotherapy.	[17]
**Neuroimmune preclinical evidence**	In the HNSCC/OSCC model, CGRP-positive sensory nerves were present in the tumor tissue; CGRP-deficient mice had smaller tumors and increased infiltration of CD4^+^, CD8^+^ T cells and NK cells.	[101]
**Human ex vivo/translational evidence**	In HNSCC patients, CD8^+^ memory T cells are sensitive to adenosine/A_2A_R-mediated chemotactic inhibition; knockdown of ADORA_2A_ restores their chemotactic capacity.	[102]
**Emerging cancer neuroscience evidence**	Tumor-invasive nociceptor neurons can promote MDSC infiltration and CD8^+^ T-cell exhaustion; this evidence comes from HNSCC and melanoma models, representing a strong mechanism but not evidence from patients experiencing radiation pain.	[103]
**Hypothesis-generating**	There is currently a lack of direct prospective clinical evidence that radiation-induced pain itself promotes immune escape/treatment resistance in HNSCC patients.	/

**Table 3:** Translational readiness of neuroimmune pain-directed strategies in head and neck cancer.

Strategy	Current Evidence	Translational Readiness
**Standard multimodal analgesia and OM supportive care**	Clinical practice and guidelines supported; current standard of supportive care [31]	Established supportive-care strategy
**taVNS + pregabalin for RRNP**	RELAX phase 2 randomized sham-controlled trial; long-term efficacy and immune effects have not been established [24]	Promising adjunctive analgesic strategy requiring further validation
**CGRP pathway inhibition**	HNSCC/OSCC studies include human sample correlation, animal model studies, and in vitro T-cell/TIL studies; rimegepant and other hypotheses are mostly repurposing hypotheses [14,104]	Candidate investigational target
**RAMP1 inhibition**	Research on the neural niche, Treg, and RAMP1 signaling mechanisms in OSCC/HNSCC; efficacy of RAMP1 blockade + anti-PD-1 in mice [105]	Preclinical immunomodulatory strategy
**A_2A_R-CGRP axis targeting**	In the OSCC model, adenosine-A_2A_R triggers CGRP release, and the A_2A_R/CGRP pathway blockade inhibits tumor growth in mice [106]	Biologically plausible but unvalidated in radiotherapy-treated HNSCC patients
**Pain-guided immunotherapy**	No validated clinical algorithm for HNSCC; prospective studies combining pain phenotype, opioid exposure, immune profiling, and radiotherapy dosimetry are needed	Research framework rather than a clinical decision tool

**Table 4:** Clinical features of different pain phenotypes in head and neck cancer.

Pain Phenotype	Typical Clinical Features	Underlying Biological State	Implications for Radiotherapy/Immunotherapy Combination
**Inflammatory/mucositis pain**	Oral pain, odynophagia, ulceration, difficulty eating	Epithelial injury, amplified inflammation, barrier disruption, increased risk of infection	Prioritize oral mucosal protection, nutritional support, local analgesia, and opioid-sparing strategies; avoid radiotherapy interruption caused by uncontrolled pain [29,107]
**Neuropathic pain**	Burning, stabbing, electric shock-like pain, numbness, allodynia	Nerve injury, neural sensitization, possible neuroimmune suppression	Prioritize gabapentin/pregabalin and neuromodulatory approaches; explore combination strategies targeting neural signaling pathways [107,110]
**Chronic/high opioid-burden pain**	Persistent long-term pain, high opioid requirement	Central sensitization, chronic inflammation, increased risk of immune impairment	Minimize opioid exposure when possible; if opioids are required during immunotherapy, potential immunologic burden should be considered, with preference for multimodal analgesia [111,112,113,114]

## Data Availability

No new data were created or analyzed in this study. Data sharing is not applicable to this article.

## Abbreviations

The following abbreviations are used in this manuscript:

A_2A_R	A_2A_ receptor
BMI	Body mass index
CAFs	Cancer-associated fibroblasts
CALR	Calreticulin
CGRP	Calcitonin gene-related peptide
DAMPs	Damage-associated molecular patterns
DCs	Dendritic cells
DLNs	Draining lymph nodes
ECM	Extracellular matrix
ENI	Elective nodal irradiation
ESCC	Esophageal squamous cell carcinoma
HIF	Hypoxia-inducible factor
HNSCC	Head and neck squamous cell carcinoma
HPV	Human papillomavirus
ICD	Immunogenic cell death
ICI	Immune checkpoint inhibitor
IFN-I	Type I interferon
iNOS	Inducible nitric oxide synthase
MDSCs	Myeloid-derived suppressor cells
MME	Morphine milligram equivalent
OM	Oral mucositis
OSCC	Oral squamous cell carcinoma
PAMORAs	Peripherally acting μ-opioid receptor antagonists
PBM	Photobiomodulation
PNI	Perineural invasion
PRRs	Pattern recognition receptors
QoL	Quality of life
RAMP1	Receptor activity-modifying protein 1
ROS	Reactive oxygen species
SP	Substance P
taVNS	Transcutaneous auricular vagus nerve stimulation
TILs	Tumor-infiltrating lymphocytes
TIME	Tumor immune microenvironment
TME	Tumor microenvironment
Tregs	Regulatory T cells
